# Systematic versus perilesional sampling in transperineal MRI-fusion biopsy: is perilesional sampling ready for prime time? A high-volume center analysis

**DOI:** 10.1186/s12957-026-04606-x

**Published:** 2026-09-23

**Authors:** Agneta Seebold, Thomas Büttner, Jörg Ellinger, Glen Kristiansen, Marit Bernhardt, Alexander Isaak, Christian Hoffmann, Julian A. Luetkens, Manuel Ritter, Philipp Krausewitz

**Affiliations:** 1 Department of Urology and Pediatric Urology, University of Bonn, University Hospital Bonn, Bonn, Germany; 2 University of Bonn, University Hospital Bonn, Institute of Pathology, Bonn, Germany; 3 University of Bonn, University Hospital Bonn, Clinic for Diagnostic and Interventional Radiology, Bonn, Germany; 4 Department of Urology, GFO Kliniken Troisdorf, Troisdorf, Germany; 5 Department of Urology, University Hospital Bonn, Venusberg-Campus 1, Bonn, 53127 Germany

**Keywords:** Prostate cancer, MRI-targeted biopsy, Perilesional sampling, Systematic biopsy, Transperineal biopsy, Risk assessment

## Abstract

**Background:**

Multiparametric MRI has improved prostate cancer (PCa) diagnostics. The current standard combines MRI-targeted fusion biopsy (TB) with 12-core systematic biopsy (SB); current guidelines allow replacing SB with perilesional sampling (PB) penumbra to maintain diagnostic performance while reducing biopsy burden.

**Methods:**

We retrospectively analyzed 314 biopsy-naive men with Prostate Imaging Reporting and Data System (PI-RADS) 3–5 lesions who underwent transperineal MRI-fusion biopsy (TB plus 12-core systematic biopsy) under local anesthesia. A simulated PB strategy was evaluated post hoc by selecting four of the twelve systematic cores located in the directly adjacent sectors to the MRI-visible lesion on the standardized PI-RADS sector map and compared with SB. The primary objective was to assess whether PB replicates the diagnostic performance of SB for detecting clinically significant PCa (csPCa; ISUP grade group ≥ 2). We also compared biopsy and prostatectomy ISUP-grading, NCCN (National Comprehensive Cancer Network) -classification and the Briganti nomogram-based lymph-node metstasis risk. Statistical analyses used R and SPSS, with chi-square or Fisher’s exact tests for categorial and t-tests for continuous variables; two-sided *p* < 0.05 was considered significant.

**Results:**

PCa was detected in 79.9% (251/314) of men, including 68.8% (216/314) with csPCa. TB alone detected 94.4% of csPCa, while SB detected 73.1%. The simulated TB + PB approach achieved 98.0% overall PCa detection and 99.1% csPCa detection (*p* = 0.48 vs. TB + SB). Concerning the impact on NCCN risk assessment, ISUP grade distribution was comparable across TB-based strategies, whereas SB alone detected fewer cancers and showed a trend towards lower ISUP grades. In the prostatectomy subgroup, ISUP grade concordance did not differ significantly between TB + SB and TB + PB (both 62.0%; *p* = 1.0). Mean Briganti scores did not differ significantly between TB + SB and TB + PB, with a minimal absolute difference of 0.17%. A 75% reduction of biopsy cores would be reached by a replacement of SB with PB. There was no significant loss of detection for csPCa (ISUP grade group 2–5; 99.1%) and no association with a loss of diagnostic yield for all ISUP grade groups. 11% reduction in detected cases was confined to ISUP grade group 1 (Supplementary Table S2).

**Conclusions:**

A TB plus 4-core PB may serve as an efficient alternative to conventional 12-core SB, without a significant reduction in diagnostic accuracy or a change in pretreatment risk assessment while markedly reducing procedural burden and resource use.

**Supplementary Information:**

The online version contains supplementary material available at https://doi.org/10.1186/s12957-026-04606-x.

## Introduction

Distinguishing clinically significant prostate cancer (csPCa) from clinically non-significant disease (nsPCa) is central to clinical decision-making and aims to ensure treatment of csPCa while minimizing overdiagnosis and overtreatment [1]. Multiparametric MRI (MRI) provides valuable diagnostic information but lacks sufficient sensitivity and specificity to serve as standalone tool, necessitating histopathological confirmation by prostate biopsy [2].The current diagnostic standard combines MRI-targeted fusion biopsy (TB) with a 12-core systematic biopsy (SB) [3]. According to the current European Guidelines, however, SB may be omitted in favor of perilesional sampling (PB) when an MRI-suspicious lesion is present [4–6].

This shift reflects growing evidence that extensive systematic sampling adds limited diagnostic value beyond targeted biopsy while increasing biopsy burden and overdiagnosis of nsPCa, particularly in patients with unilateral MRI-visible lesions. Previous studies suggest that ipsilateral and perilesional sampling captures most csPCa missed by targeted biopsy alone [7, 8]. Moreover, it was demonstrated that the detection rate of csPCa is higher using perilesional sampling beyond the index lesion in the immediate lesion environment [7, 8].

Additionally, Hagens et al. showed that a targeted plus perilesional approach (TB + PB) achieves cancer detection rates comparable to TB + SB, while requiring fewer cores and reducing overdiagnosis of indolent International Society of Urological Pathology 1 (ISUP 1) nsPCa [9]. However, the optimal number and spatial distribution of perilesional biopsies remain to be determined. Existing studies have used heterogeneous sampling strategies; for instance, Hagens et al. performed seven perilesional cores, highlighting the need for methodological standardization in this emerging approach. Moreover, it remains unclear how an altered biopsy scheme may influence PCa risk stratification, given that established models - such as the NCCN classification and the Briganti nomogram - are fundamentally based on the number of systematic core biopsies [10]. The objective of this study was to evaluate the diagnostic yield of a 12-core systematic versus a simulated 4-core perilesional scheme within transperineal MRI-fusion biopsy in our institutional cohort.

## Materials and methods

This retrospective study included 314 biopsy-naive men with Prostate Imaging Reporting and Data System (PI-RADS) 3–5 lesions on prostate MRI. All underwent transperineal MRI-fusion biopsy under local anesthesia at the Department of Urology and Pediatric Urology, University Hospital Bonn, Bonn, Germany. Both internal and external imaging were permitted. Biopsies were performed using an MRI-ultrasound fusion system (KOELIS Trinity^®^, organ-based fusion Germany). All patients underwent TB of MRI-identified lesions in combination with an institutionally standardized 12-core systematic sampling.

**Fig. 1:**
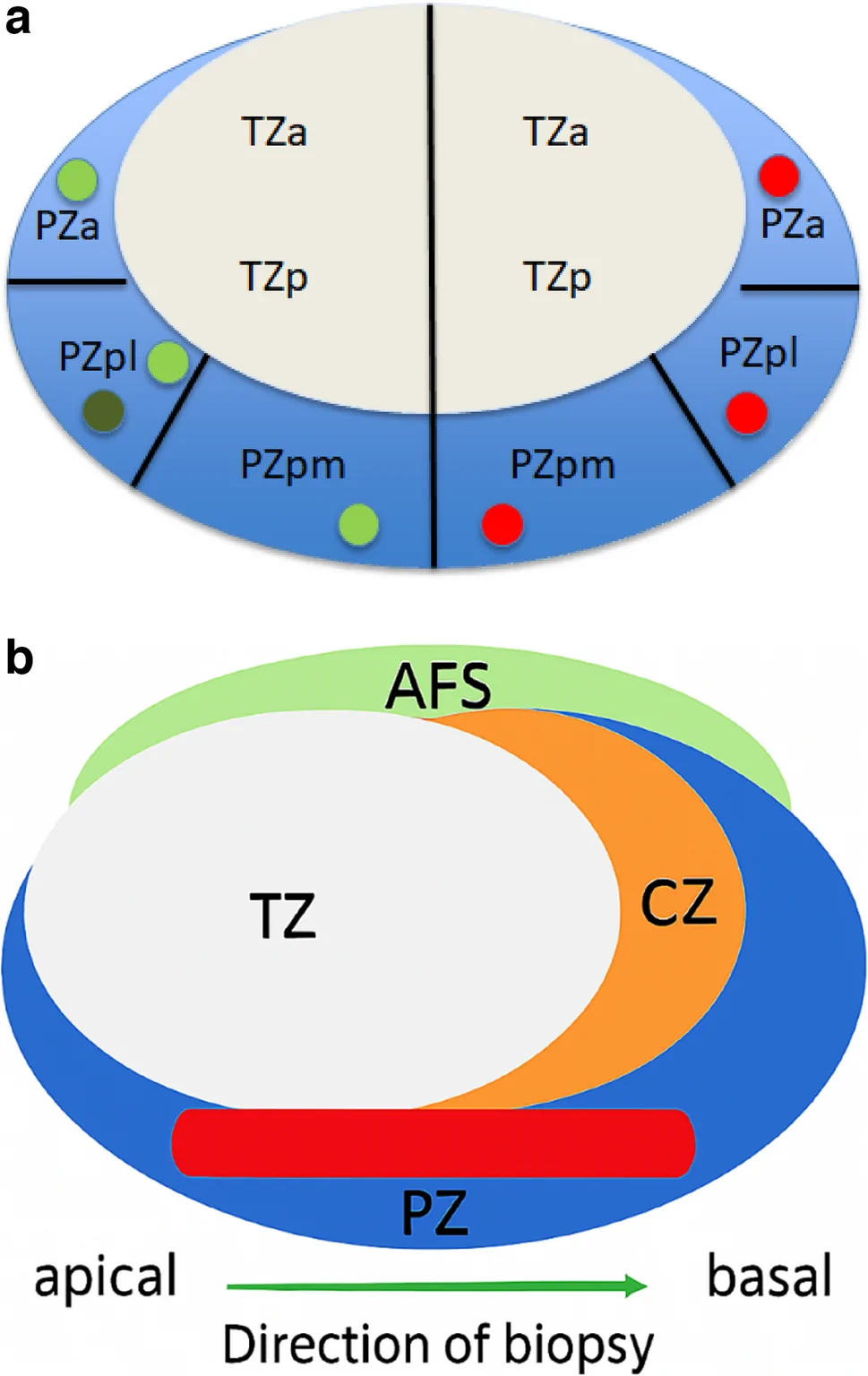
**a**: Transverse view: PI-RADS sector map illustrating the biopsy approach. The dark green dot marks the MRI-targeted lesion (target). Simulated perilesional biopsies are performed within the same sector and the directly adjacent sectors (light green dots). Red dots: additional core of SB approach. **b**: Schematic sagittal view: Prostate in the supine patient position as visualized during transrectal ultrasound and MRI-US fusion procedures; the prostatic base is shown on the right and the apex on the left. The red bar indicates the biopsy sampling zone. This view (sagittal) illustrates the cranio-caudal /Base-apex) axis in order to distinguish it from the anterior-posterior view in **a**

We predefined three cores per target lesion as number of TB cores and 12 cores for SB in accordance with our institutional standard. SB cores were obtained according to a predefined sector-based template, as illustrated in Fig. 1, encompassing the peripheral lateral, peripheral medial, and peripheral anterior zones at the apical, mid-gland, and basal levels of the prostate. Each of the 12 SB-cores was assigned to its corresponding PI-RADS sector map location to ensure anatomical consistency and reproducibility of sampling.

To evaluate a perilesional biopsy strategy in comparison with the SB approach, we retrospectively simulated perilesional sampling by selecting the systematic cores located in the same sector and in the sectors immediately adjacent to the MRI-positive lesion. For single, single-sector lesions, this corresponded to the four systematic cores located in the sector directly cranial and the sector directly caudal to the target sector on the PI-RADS sector map (i.e., along the base-apex axis of the lesion, rather than in the anterior-posterior plane; Fig. 1a-b). As PB cores were a post-hoc-selected subset of the already-acquired 12-core SB template rather than a separately performed biopsy, needle trajectory and sampling depth for each PB core were identical to those of the corresponding systematic core described above. Of the 314 target lesions, 34 (10.8%) spanned more than one PI-RADS sector; in these cases, perilesional cores were obtained within the lesion’s own sector(s) and/or the immediately adjacent sector(s), consistent with the sector-based definition applied to single-sector lesions. All samples were evaluated histologically in the Institute of Pathology of the University of Bonn, which serves as a national tertiary reference center for genite-urinary pathology, following established guidelines. CsPCa was defined as ISUP grade group ≥ 2. Detection rates and histopathologic grading were compared across biopsy methods. Tumor distribution was classified as focal, multifocal or global, based on the number and pattern of MRI-visible target lesions per patient. Focal was defined as one single MRI-visible target lesion, multifocal as two or more spatially discrete MRI-visible target lesion, separated by intervening normal-appearing tissue and global as diffuse tumor involvement extending across multiple sectors without clearly separable individual lesions.

In 79 patients (25.1%) who subsequently underwent radical prostatectomy, ISUP grading on biopsy was systematically compared with the final prostatectomy specimen to determine concordance between biopsy and definitive pathology.

Statistical analyses were performed using RStudio v2025.05.1 + 513 and SPSS. Categorical variables were compared with chi-square or Fisher’s exact tests; continuous variables with t-tests. McNemar’s tests were used to compare diagnostic accuracy between biopsy modalities. Odds ratios assessed associations between preclinical variables (Prostate specific antigen (PSA), digital rectal examination (DRE), transrectal ultrasound (TRUS), PI-RADS score, and csPCa detection. Logistic regression identified predictors based on these four pre-biopsy parameters, independent of biopsy approach. A two-sided p-value < 0.05 was considered statistically significant. For the pre-specified primary endpoint (csPCa detection, TB + SB vs. TB + PB, overall cohort), the nominal p-value is reported unadjusted.

For the secondary and subgroup comparisons (PI-RADS-stratified detection rates and pairwise biopsy-strategy comparisons beyond the primary endpoint), the Benjamini-Hochberg procedure was applied to control the false discovery rate, and both nominal and FDR-adjusted p-values are reported in Supplementary Table S1.

The Briganti Nomogram (2019 version) was used to estimate the individual risk of lymph node invasion (LNI) in PCa patients [10]. The nomogram thereby calculates the probability of pelvic lymph node metastasis and supports the decision-making process regarding the indication for extended pelvic lymph node dissection, using a 5% risk threshold.

Preoperative variables of all PCa patients including PSA level, clinical tumor stage, ISUP grade, and the percentage of positive biopsy cores (TB + SB) were entered into the model [10]. For the perilesional biopsy approach, we adapted the Nomogram by entering the percentage of positive biopsy cores of TB + PB. Next, we performed a descriptive comparison on pretreatment risk stratification for LNI between the TB + SB and TB + PB approach.

We also simulated the NCCN-risk-classification for the different biopsy approaches. The TB + SB results represent the reference standard derived from the actual clinical biopsy scheme, whereas the TB-only and TB + PB results are based on the simulation biopsy, reflecting how the same patients would have been risk-classified if only the respective reduced biopsy information had been available. Apparent increases in category-specific case numbers therefore reflect risk migration rather than discrepancies in patient counts.

## Results (Table 1)

**Table 1:** Cohort characteristics

Characteristic	Value (Percentage)
Study participants	314
Median patient age	68 years (IQR 62.1–74.7)
Median prostate volume	45.0 ml (IQR 35.0–64.0 ml; *n* = 314)
PCa detection	251 (79.9%)
csPCa detection	216 (68.8%)
nsPCa detection	35 (11.1%)
Institutional MRI (quality-controlled protocol)	190 (60.5%)
External MRI	124 (39.5%)
3 Tesla MRI	223 (71.0%)
1,5 Tesla MRI	91 (29.0%)
PI-RADS Score	PI-RADS 3: 57 cases (18.2%)
	PI-RADS 4: 149 cases (47.5%)
	PI-RADS 5: 109 cases (34.7%)
Median lesion diameter (MRI)	11.4 mm (IQR 7.0–18.3 mm)
Lesions spanning > 1 PI-RADS sector	34/314 (10.8%)
Suspicious DRE	108/314 (34.4%)
Average PSA	9.83 ng/ml (IQR 5.4–10.2)
NCCN Classification	35 Low risk cases (14.0%)
	107 intermediate favorable cases (42.6%)
	46 intermediate unfavorable cases (18.3%)
	63 high risk cases (25.1%)
PCa-bearing biopsy cores overall	1047/3492 (30.0%)
PCa-bearing biopsy cores SB	841/3780 (22.3%)
PCa-bearing biopsy cores PB	687/ 1506 (46.0%)
PCa-bearing biopsy cores TB	681/900 (76.0%)

*PCa* Prostate cancer, *csPCa* clinically significant prostate cancer, *nsPCa* clinically non-significant prostate cancer, *MRI* Magnetic resonance imaging, *PI-RADS* Prostate Imaging Reporting and Data System, *DRE* Digital rectal examination, *PSA* Prostate-specific antigen, *NCCN* National Comprehensive Cancer Network, *SB* Systematic biopsies, *PB* Perilesional biopsies

### Cancer detection rates by biopsy method

Across the entire cohort, 251 men (79.9%) had PCa, with 216 csPCa (68.8%). Overall, 30.0% of biopsy cores were PCa-bearing. We selected for the simulated reduced biopsy sampling systematic cores located in the same sector and in the sectors immediately adjacent to the MRI-positive lesion, yielding a mean of 4.2 cores per patient (median 4; IQR 4–5), (Fig. 1a). In SB, 22.3% of cores were positive, whereas TB showed tumor in 76.0% of cores. In PB, 46.0% cores were positive. Thus, the proportion of positive cores was 53.7% points higher in TB compared with SB, 30.0 points higher in TB compared with PB, and 23.7 points higher in PB compared with SB (all *p* < 0.001).

TB alone detected 90.4% of all PCa cases and 94.4% of csPCa. SB alone detected 84.1% of all PCa cases and 73.1% of csPCa. PB alone detected PCa in 78.1% of cases and csPCa in 69.9%.

The perilesional biopsy approach (TB + PB) detected 99.1% of csPCa cases identified by the combined TB + SB strategy; Table 2. TB demonstrated superior csPCa detection in pairwise comparisons to SB (*p* < 0.001) and PB (*p* < 0.001), identifying significantly more cancers (McNemar’s test). SB also detected significantly more sPCa than PB alone, with an absolute difference in detection rate of 3.2% (*p* = 0.016). In contrast, when combined with TB, the detection rates were almost identical (TB + SB vs. TB + PB), differing by only 0.5% (*p* = 0.48).

**Table 2:** Cancer detection rates for PCa and csPCa for the different biopsy strategies

Biopsy strategy	Detection Rate PCa	Detection Rate csPCa
TB alone	90.4% (227/251)	94.4% (204/216)
SB alone	84.1% (211/251)	73.1% (158/216)
PB alone	78.1% (196/251)	69.9% (151/216)
TB + SB	100%	100%
TB + PB	98.0% (246/251)	99.1% (214/216)

**Fig. 2:**
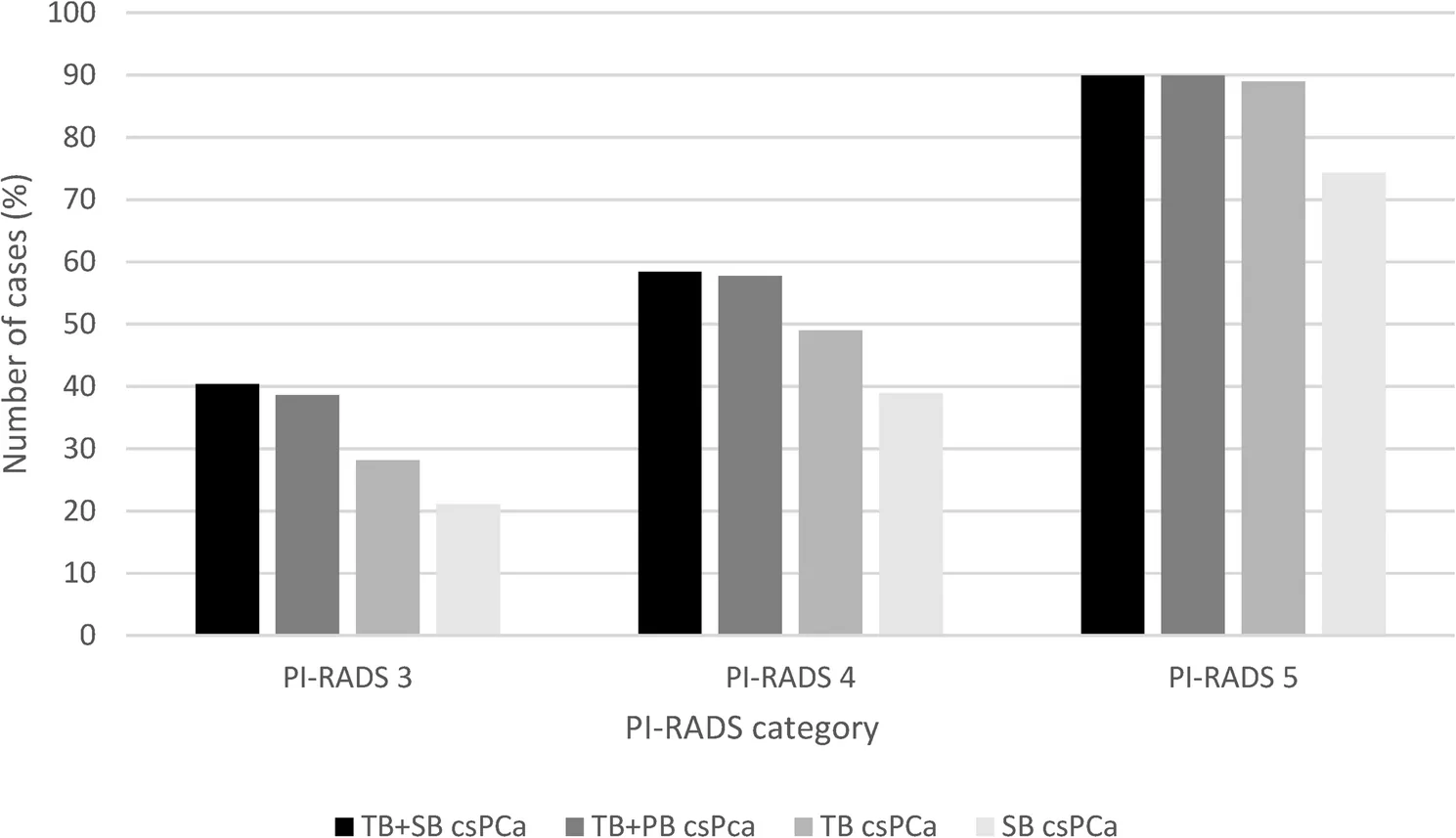
Cancer detection rates (CDRs) for csPCa across PI-RADS categories 3–5, stratified by biopsy approach: combined biopsy (TB + SB), reduced perilesional sampling approach (TB + PB), targeted biopsy (TB), and systematic biopsy (SB)

Across the different biopsy approaches the PI-RADS-stratified detection of csPCa varied markedly (Fig. 2): in PI-RADS 3 lesions, detection was 40.4% (23/57; TB + SB), 38.6% (22/57; TB + PB), 28.1% (16/57; TB), and 21.1% (12/57; SB); in PI-RADS 4, rates were 63.8% (95/149; TB + SB), 63.1% (94/149; TB + PB), 54.4% (81/149; TB), and 43.6% (65/149; SB); and in PI-RADS 5, detection reached 89.9% (98/109; TB + SB), 89.9% (98/109; TB + PB), 89.0% (97/109; TB), and 74.3% (81/109; SB). Considering the PI-RADS dependent detection rates above (McNemar’s test), significant differences were demonstrated for TB + SB versus SB in PI-RADS 3 (*p* = 0.003), PI-RADS 4 (*p* < 0.001) and PI-RADS 5 (*p* < 0.001). Differences were also significant concerning TB + SB vs. TB alone in PI-RADS 3 (*p* = 0.016), PI-RADS 4 (*p* < 0.001) but not concerning PI-RADS 5 (*p* = 1.0), whereas detection rates among TB + SB, TB + PB and TB alone did not differ significantly (all *p* > 0.05), Fig. 2.

### Overdiagnosis and missed diagnosis

In the overall cohort, combined targeted and perilesional 4-core biopsy achieved a PCa detection rate of 98.0% and a csPCa detection rate of 99.1%, identifying 98.4% (62/63) of high-risk PCa (defined as ISUP grade group 4 or 5); consequently, the missed diagnosis rates of csPCa and high-risk PCa with TB + PB compared with the standard TB + SB approach were 0.9% (2/216) and 1.6% (1/63), respectively. On the other hand, omitting SB in favor of a 4-core PB in TB-positive cases would have reduced the number of additional cores by 75% (12 systematic biopsies to 4).

In 80/251 (31.9%) men, tumor foci were detected outside the MRI-index lesion and beyond the perilesional penumbra, defined as the PI-RADS sectors located cranio-caudally adjacent to the MRI lesion. Of these, 37.5% were nsPCa (30/80), 62.5% were csPCa (50/80), and 17.5% high-risk PCa (14/80). Of note, in only one of these cases, csPCa was detected exclusively outside both the MRI-target lesion and the penumbra.

Upgrading due to tumor detection outside the target lesion and its penumbra occurred in 10.0% of patients (8/80). The upgrading rate varied depending on the PI-RADS score: among PI-RADS 3 lesions, 2 of 9 cases were upgraded (22.2%); in PI-RADS 4 lesions, 3 of 31 cases (9.7%) were upgraded; and in PI-RADS 5 lesions, 3 of 42 cases (7.1%) were upgraded.

In the subgroup of patients with negative TB, SB alone detected PCa in 9.6% (24/251), of which half were clinically significant (12/24), corresponding to 5.6% of all csPCa cases. Among those 24 TB-negative patients, PB detected PCa in 79.2% (19/24), including 91.7% (11/12) of csPCa and 75.0% (3/4) of high-risk disease. Consequently, replacing SB with PB in this subgroup would have reduced diagnosis of nsPCa by 20.8% (5 cases), at the cost of delayed detection of a single csPCa case (ISUP grade group 4).

In the entire cohort, SB upgraded targeted biopsy findings to csPCa in 8.8% (22/251) of patients, including 7.2% (18/251) upgraded to high-risk disease. In comparison, PB resulted in upgrading to csPCa in 8.0% (20/251), including 6.4% (16/251) to high-risk PCa.

Accordingly, upgrading to csPCa (8.8% vs. 8.0%, *p* = 1.0) and high-risk disease (7.2% vs. 6.4%, *p* = 0.48) was comparable between approaches (McNemar’s test).

Concordant csPCa detection—defined as agreement in maximum ISUP grade group between biopsy approaches—occurred also at comparable rates for TB versus SB (40.7%, 88/216) and TB versus PB (39.4%, 85/216).

### Clinical implications: tumor grading and risk of nodal spread

NsPCa was diagnosed in 35 patients (11.1% of the cohort). TB identified 23 of those 35 cases, while TB + PB detected 31; SB alone would have identified 12/35 cases exclusively in TB-negative patients. Consequently, omitting SB would have reduced the number of men diagnosed with non-significant disease alone by 12, whereas use of TB + PB would have reduced nsPCa diagnoses by four, corresponding to an 11% reduction in ISUP 1 overdiagnosis using PB rather than SB.

Stratification by NCCN risk groups demonstrated comparable grading performance among TB-based strategies. TB + SB identified 251 prostate cancer cases, including 35 low-risk, 107 favorable intermediate-risk, 46 unfavorable intermediate-risk, and 63 high-risk tumors. TB alone detected 90.4% of cancers identified by TB + SB, with statistically significant underdetection across all risk categories (McNemar’s test), most pronounced for unfavorable intermediate- and high-risk disease (19.6% less for intermediate unfavorable and 28.6% less for high risk; *p* < 0.001); Table 3.

**Table 3:** NCCN risk group distribution across TB-based strategies

NCCN Biopsy Strategy	All PCa	Low risk	Intermediate favorable risk	Intermediate unfavorable risk	High risk
TB + SB	251	35	107	46	63
TB	227	33	112	37	45
TB + PB	246	32	107	45	62

TB + PB achieved near-complete concordance with TB + SB with only marginal differences across risk strata. Overall agreement was 98.0% (246/251), corresponding to a kappa of 0.95 (95% CI 0.91–0.99). In contrast, SB alone detected substantially fewer cancers and demonstrated.

systematic grade migration toward lower ISUP categories, characterized by an overrepresentation of ISUP 1 and a marked underdetection of ISUP 3 tumors; Supplementary Table 2.

In the prostatectomy subgroup (*n* = 79), concordance between biopsy and prostatectomy ISUP grade group was 62.0% (49/79) for both biopsy approaches (TB + SB and TB + PB). Concordance rates were lower for TB alone (58.2%; 46/79) and lowest for SB alone (39.2%; 31/79), underscoring the superior grading accuracy of the combined approaches.

With respect to tumor extension, focal tumors (*n* = 57) showed 6 upgradings (10.5%), multifocal tumors (*n* = 16) had one upgrading (6.2%), and global tumors (*n* = 9) had one upgrading (11.1%) detected by systematic sampling (SB or PB).

The median Briganti score was 14.0% (IQR 3.25–22.25) when calculated from all TB + SB cores and 13.0% (IQR 3.00-25.75) when calculated using only TB + PB cores. In paired analysis, the median difference was minimal (+ 0.17% points) and not statistically significant (*p* = 0.58, Wilcoxon signed-rank test).

## Discussion

In this retrospective analysis, we found that combining TB with a reduced 4-core perilesional sampling scheme did not differ significantly in csPCa detection rates from the current standard of care using TB plus 12-core SB. Specifically, TB + PB detected 99.1% of csPCa and 98.4% of high-risk disease, while reducing the additional number of biopsy cores by 75%. The very low rate of missed sPCa underscores the diagnostic safety and efficiency of this focused four-core perilesional strategy.

Importantly, TB alone identified the vast majority of csPCa, while perilesional sampling appeared to capture most of the remaining csPCa and high-risk tumors missed by TB. In contrast, extensive sampling by SB contributed marginal additional csPCa cases and was more frequently associated with the diagnosis of ISUP 1 PCa. These findings indicate that reducing the extent of systematic sampling to a perilesional 4-core approach could markedly lower biopsy burden and procedural costs-both human and material—while maintaining diagnostic reliability, in line with the conceptual framework of MRI-defined penumbra sampling proposed previously [7, 9]. This streamlined biopsy protocol may thus represent a pragmatic and safe alternative to extensive systematic biopsy in MRI-guided PCa diagnosis.

This argument is further supported by our comparison between biopsy and prostatectomy ISUP grade groups, where no difference in concordance of the ISUP grading could be shown between the 12-core SB and 4-core PB approach. Moreover, upgrading was observed at similar rates comparing focal, global and multifocal tumors. In line with previous findings, both combined approaches showed an improved cancer detection rate compared to a TB-alone setting, underscoring the continued importance of combining targeted biopsy with additional sampling [9].

The current results support a reduced, anatomically guided biopsy strategy that prioritizes targeted and ipsilateral perilesional cores over whole-gland systematic biopsy, thereby maintaining high diagnostic accuracy while minimizing patient burden, lowering costs, and reducing overdiagnosis of nsPCa. Brisbane et al. proposed a PI-RADS-based approach defining a 1 cm “penumbra” around the index lesion, whereas our study employed a sector-based strategy using the standardized PI-RADS map [7]. They showed that 90% of csPCa-positive biopsy cores were located within 10 mm of the MRI target, with diagnostic yield declining sharply beyond this margin. Importantly, the spatial extent of this penumbra was PI-RADS dependent, encompassing approximately 5 mm for PI-RADS 5 lesions, 12 mm for PI-RADS 4 lesions, and up to 16 mm for PI-RADS 3 lesions [7]. Hagens et al. reported that notably, when relying solely on additional perilesional sampling, missed csPCa occurred most frequently in men with PI-RADS 3 lesions (16.7%), compared with substantially lower miss rates in PI-RADS 4(2.5%) and PI-RADS 5 lesions (2.0%) [9]. Our results corroborate these findings and add an additional perspective. Specifically, the incremental benefit of systematic sampling over the TB-only approach was confined to PI-RADS 3–4 lesions, whereas in PI-RADS 5 lesions TB alone demonstrated comparable diagnostic accuracy for csPCa. Notably, in our study the added value of SB over a reduced PB was marginal for PI-RADS 3 and 4 lesions. Overall, csPCa detection did not differ significantly among TB + SB, TB + PB, and TB across PI-RADS categories.

In line with Noujeim et al., who also demonstrated that restricting additional sampling to the perilesional zone achieves csPCa detection rates comparable to the combined TB + SB approach, our findings indicate that replacing SB with PB avoids the detection of some low-risk disease located at more distant sites [8]. In this regard, studies showed that cancer detection plateaued at approximately 90% once more than five TB cores were obtained, while additional systematic biopsy provided only marginal gains at the cost of increased detection of ISUP 1 disease and higher biopsy-related morbidity [11]. Consequently, multiple groups have reported that the incremental yield of additional sampling is largely confined to tissue immediately adjacent to the index lesion [12, 13].

Perilesional biopsy schemes using two to seven additional cores have been shown to significantly improve csPCa detection compared with TB alone, while avoiding the low-yield contralateral systematic cores [14, 15]. Hagens et al. reported in a systematic review equivalent csPCa detection for TB + PB compared to TB + SB, with a csPCa detection difference of 3%, while reducing the number of total biopsy cores by more than one third (total amount of 7 biopsy cores (each 3–5 TB plus 2–4 PB). Moreover, ipsilateral sampling strategies (each 6 ipsilateral systematic biopsy cores) yielded csPCa sensitivities of 96–99% in cohorts by Bryk, Freifeld, Jager and Ruan, whereas contralateral systematic biopsy contributed only marginal to csPCa detection and primarily increased overdiagnosis [13, 14, 16, 17]. In the current analysis csPCa detection differed by only 1%, without reaching statistical significance, and a reduction of nsPCa by 11%. Hence, together with previously published retrospective and meta-analytical data, our results provide further rationale for redefining systematic biopsy protocols in the context of MRI-targeted prostate biopsy, reinforcing the principle that “less is more” [8, 16, 18, 19].

Our findings should also be interpreted alongside the recent multicentre study by Cannoletta et al., who compared TB + PB with TB + SB for csPCa detection in 2,852 patients with PI-RADS ≥ 3 lesions across three tertiary referral centres [20]. In their cohort, TB + SB detected significantly more csPCa than TB + PB overall (50% vs. 45%, *p* = 0.03), an effect driven primarily by PI-RADS 3 lesions (26% vs. 21%, *p* = 0.02) and patients under active surveillance (46% vs. 40%, *p* = 0.04), whereas no significant difference was observed in PI-RADS 4 (53% vs. 50%) or PI-RADS 5 lesions (76% vs. 75%), nor in biopsy-naïve or previous-negative-biopsy patients. Our PI-RADS-stratified results are concordant with these findings for PI-RADS 4 and 5 lesions (63.8% vs. 63.1% and 89.9% vs. 89.9%, respectively), but diverge for PI-RADS 3 lesions, where we observed no significant difference between TB + SB and TB + PB (40.4% vs. 38.6%, *p* = 1.0). This discrepancy most likely reflects the markedly smaller size of our PI-RADS 3 subgroup (*n* = 57) relative to the multicentre cohort, and the absence of active surveillance patients—a subgroup in which Cannoletta et al. identified a specific detection disadvantage for TB + PB—from our biopsy-naïve cohort. Differences in the anatomical definition of the perilesional sampling region may further contribute to this divergence at lower PI-RADS categories, where lesion conspicuity is inherently reduced.

Thereby, a purely combined lesion and penumbra-guided biopsy strategy appears to confer a very low risk of missing csPCa, as in the current study only a single case harbored csPCa exclusively outside the index lesion and its penumbra. Conversely, additional tumor foci were identified outside the target lesion and its penumbra in 32% of PCa patients, of which one third were ISUP grade 1 and two thirds represented csPCa, underscoring limitations in accurately capturing csPCa extent with a reduced TB + PB approach. For the purpose of this analysis, we defined these extra-penumbral foci - located outside both the target lesion and its ipsilateral perilesional sectors - as contralateral disease. Of the 216 csPCa cases in our cohort, 50 (23.1%) harbored at least one contralateral tumor focus that would not have been sampled by TB + PB; however, only a single case (0.5%) had csPCa located exclusively contralaterally, meaning it would have been entirely missed by the TB + PB strategy.

Consequently, perilesional sampling cannot overcome the well-recognized limitations of MRI in detecting secondary csPCa foci, as demonstrated in whole-mount correlation studies, although it may mitigate targeting errors [21, 22]. We consider this localization limitation - rather than csPCa detection per se - to be the principal clinical limitation of the TB + PB approach; because perilesional sampling is confined to the sectors adjacent to the index lesion, it cannot reliably identify csPCa located contralaterally, which is directly relevant for surgical planning, including decisions on nerve-sparing during radical prostatectomy and candidate selection for focal therapy. Intraoperative frozen-section-guided nerve-sparing has been shown to improve functional outcomes without compromising oncological safety and could, in principle, mitigate the impact of incomplete biopsy-based tumor mapping on nerve-sparing decisions [19]. However, this technique is not routinely available in most centers owing to its substantial additional cost relative to obtaining 6–8 further systematic cores, and in centers without access to it, comprehensive biopsy-based tumor localization - rather than csPCa detection alone - remains directly relevant to surgical planning. Accordingly, perilesional biopsy should not be considered a replacement for comprehensive risk assessment, but rather as a focused strategy that substantially reduces biopsy burden while preserving very high diagnostic accuracy and safety. Yet, based on the present data, a reduced approach may allow for comparable pretreatment risk assessment with respect to tumor grading and the likelihood of nodal involvement. The Briganti score, which is traditionally calculated based on cancer burden derived from 12-core systematic biopsy, did not differ significantly when calculated from TB + SB cores compared with a TB + PB-based approach. This suggests that using perilesional biopsy cores may not necessarily alter nodal risk stratification by established risk models in a clinically meaningful way. Similarly, NCCN risk assessment based on ISUP grade distribution showed no significant difference in risk categorization across TB-based strategies. Taken together, these findings indicate that TB-focused biopsy approaches may preserve the integrity of established pretreatment risk models while calling into question the incremental clinical value of high-core systematic sampling. Our findings are in line with the recently published ProBIOPSY study, which harmonized key elements of a targeted-plus-perilesional biopsy scheme and considered it sufficient for most aspects of local treatment planning, while noting that additional contralateral sampling may still be required for specific indications such as focal therapy candidate selection - consistent with the localization limitation discussed above [20].

Our study has limitations. First the retrospective design and the limited cohort size constrain the generalizability of our findings. Additionally, the study was neither designed nor powered to formally assess statistical equivalence or non-inferiority between the biopsy schemes. Furthermore, our detection rates should be interpreted in the context of the referral population undergoing biopsy. In the recent 23-year follow-up of the ERSPC screening trial, only 24% of biopsies performed after a positive PSA screen confirmed prostate cancer [23], whereas our cohort was pre-selected based on MRI-visible PI-RADS 3–5 lesions, yielding a substantially higher detection rate (79.9%); these detection rates are not directly comparable and reflect differences in patient selection and diagnostic pathways between population-based PSA-screening-triggered biopsy and MRI-triaged biopsy cohorts. Looking forward, the recently published European Cancer Prevention Organization consensus endorses structured risk-adapted PSA testing for prostate cancer as part of a broader European roadmap toward precision screening, which may further shape the composition of future biopsy-referred cohorts and, consequently, reported detection rates [24].Second, the institutional 12-core standardized approach may introduce some inaccuracy in the anatomical allocation of cores to the corresponding PI-RADS sectors. MRI interpretation, including lesion localisation, PI-RADS sector assignment, and lesion size assessment, was performed by at least two experienced radiologists; however, these assessments were not performed independently and blinded to each other, and no formal inter-rater reliability analysis (e.g., Cohen’s kappa) was performed. For an expanded targeted approach comprising both the target lesion and its perilesional penumbra, it will be important for future trials to also account for radiological quality, including blinded assessment and, where possible, central review.

Next, the tested nomogram and the NCCN classification system was not validated for a reduced systematic sampling scheme, hence comparisons yield a methodical bias.

Furthermore, pathological lymph node status from extended pelvic lymph node dissection was not systematically available for all patients, precluding a direct comparison of the discriminative accuracy (e.g., area under the receiver operating characteristic curve) of the standard and TB + PB-based Briganti nomogram against a pathological ground truth; this should be addressed by future studies with systematic nodal status ascertainment.

Importantly, the PB approach evaluated in this study was a simulation based on a subset of systematic biopsy cores rather than a prospectively performed stand-alone PB strategy in an independent PB protocol performed with a dedicated sampling intention. A prospectively designed PB-only strategy might have yielded different results and should be evaluated in future studies.

A limitation of our approach is that perilesional biopsy cores were defined post hoc based on the anatomical sectors immediately adjacent to the index lesion rather than on the actual spatial extent of the lesion. Thus, lesions extending across multiple PI-RADS sectors may not have been fully captured by the predefined perilesional sampling strategy.

Similarly, because PB sector assignment is anatomically pre-defined and independent of the TB pathology result, our exploratory subgroup analysis in TB-negative patients is not subject to selection bias in core assignment; nonetheless, as a retrospective, post-hoc subgroup analysis, this finding should be regarded as hypothesis-generating rather than as validation of an independently, prospectively performed PB-only strategy in TB-negative men. Future prospective studies should therefore consider lesion-specific factors, including lesion size and location, to establish an optimized and standardized perilesional sampling strategy. In this context, lesion size and extent also depend on the quality of radiological assessment; future trials employing an expanded target-plus-penumbra biopsy approach should therefore incorporate blinded and, ideally, centralized radiological review, both of which could not be implemented within the retrospective design of the present study.

Nevertheless, we evaluated, for the first time, how a reduced 4-core perilesional PIRADS-2 sector-map-based additional biopsy could influence the clinical interpretation of biopsy results prior to definitive treatment with concerns to risk assessment of tumor grading, tumor distribution and nodal spread. Our results suggest that adopting a targeted 4-core perilesional strategy has potential to streamline biopsy protocols without compromising diagnostic accuracy. Future prospective research should focus on optimizing the spatial placement of perilesional cores, determining the minimum number of cores required to maintain diagnostic safety, assessing patient reported outcomes and developing new nomograms—or adapting existing ones—to guide treatment decisions after penumbra-based sampling. Given that our PB strategy was retrospectively simulated rather than prospectively performed, these findings should be regarded as hypothesis-generating rather than practice-changing, and confirmation in an independently, prospectively performed PB protocol is needed before this approach can be considered for routine clinical adoption. This is consistent with the broader ProBIOPSY consensus, which likewise underscores the considerable potential of a combined targeted-plus-perilesional strategy while acknowledging that the optimal biopsy framework - including the number, location, and definition of perilesional cores - has yet to be established [25].

## Conclusions

Our data supports a reduced perilesional 4-core systematic biopsy approach as an adjunct to MRI targeted biopsy as an efficient alternative to the conventional 12-core multifocal systematic biopsy. In the current cohort, this approach reduced the number of biopsy cores without a statistically significant reduction in csPCa detection, highlighting its potential to streamline prostate biopsy while maintaining diagnostic yield. From a patient perspective, reducing the number of systematic cores has the potential to decrease procedure-related discomfort and may improve overall tolerability and acceptance of prostate biopsy. Given the retrospective, simulated nature of the perilesional biopsy strategy evaluated here, these findings should be considered hypothesis-generating. Prospective studies are warranted to validate these findings and to establish optimized penumbra-based protocols.

## Supplementary Information

Supplementary Material 1.

## Data Availability

No datasets were generated or analysed during the current study.

## Abbreviations

csPCa: Clinically significant prostate cancer
DRE: Digital rectal examination
ISUP: International Society of Urological Pathology
LNI: Lymph node invasion
MRI: Magnetic resonance imaging
NCCN: National Comprehensixe Cancer Network
nsPCa: Clinically non-significant prostate cancer
PB: Perilesional Biopsy
PCa: Prostate cancer
PI-RADS: Prostate Imaging Reporting and Data System
PSA: Prostate-specific antigen
SB: Systematic Biopsy
TB: Targeted Biopsy
TRUS: Transrectal ultrasound
