# Supplementary material for: Systematic versus perilesional sampling in transperineal MRI-fusion biopsy: is perilesional sampling ready for prime time? A high-volume center analysis
Source: World J Surg Oncol. 2026 Sep 23;24:378. doi: 10.1186/s12957-026-04606-x (PMC13599324; doi:10.1186/s12957-026-04606-x)
Supplement: Supplementary file 1 [file 12957_2026_4606_MOESM1_ESM.docx]

**Supplementary Table S1. Detection rates by biopsy strategy, overall and stratified by PI-RADS category, with statistical comparisons.**
The pre-specified primary endpoint (overall sPCa detection, TB + SB vs. TB + PB) is reported unadjusted. For all other (secondary/subgroup) comparisons, the Benjamini-Hochberg procedure was applied to control the false discqvery rate across this family of 15 comparisons; both nominal and FDR-adjusted p-values are shown. All conclusions of statistical significance are unchanged after FDR adjustment.

*All pairwise comparisons were performed using McNemar's test for paired diagnostic-accuracy data, consistent with the Methods section.*

| **Comparison** | **csPCa detection n/N (%)** | **p-value (csPCa, McNemar)** | **BH-adjusted p (FDR)** |
| --- | --- | --- | --- |
| Overall cohort (N = 314; PCa = 251; csPCa = 216) |  |  |  |
| TB alone (vs TB + SB) | 204/216 (94.4%) | 0.001 | < 0.001 |
| SB alone (vs TB + SB) | 158/216 (73.1%) | < 0.001 | < 0.001 |
| PB alone (vs TB + SB) | 151/216 (69.9%) | < 0.001 | < 0.001 |
| TB + SB (reference) | 216/216 (100%) | reference | reference |
| TB + PB (vs TB + SB) | 214/216 (99.1%) | 0.48 | primary endpoint (unadjusted) |
| TB vs SB | — | < 0.001 | < 0.001 |
| TB vs PB | — | < 0.001 | < 0.001 |
| SB vs PB | — | 0.016 | 0.032 |
| PI-RADS 3 (n = 57) |  |  |  |
| TB + SB (reference) | 23/57 (40.4%) | reference | reference |
| TB + PB (vs TB + SB) | 22/57 (38.6%) | 1.0 | 1.0 |
| TB (vs TB + SB) | 16/57 (28.1%) | 0.016 | 0.032 |
| SB (vs TB + SB) | 12/57 (21.1%) | 0.003 | 0.004 |
| PI-RADS 4 (n = 149) |  |  |  |
| TB + SB (reference) | 95/149 (63.8%) | reference | reference |
| TB + PB (vs TB + SB) | 94/149 (63.1%) | 1.0 | 1.0 |
| TB (vs TB + SB) | 81/149 (54.4%) | < 0.001 | < 0.001 |
| SB (vs TB + SB) | 65/149 (43.6%) | < 0.001 | < 0.001 |
| PI-RADS 5 (n = 109) |  |  |  |
| TB + SB (reference) | 98/109 (89.9%) | reference | reference |
| TB + PB (vs TB + SB) | 98/109 (89.9%) | 1.0 | 1.0 |
| TB (vs TB + SB) | 97/109 (89.0%) | 1.0 | 1.0 |
| SB (vs TB + SB) | 81/109 (74.3%) | < 0.001 | < 0.001 |

**Supplementary Table S2. PCa detection and undiagnosed cases by ISUP grade group (reference standard: TB + SB), comparing TB alone and TB + PB.**
*Reference ISUP grade group is defined by the combined TB + SB result (n = 251 PCa cases; 314 biopsy-naive men total). "Undiagnosed" denotes patients in whom the given strategy did not identify any* carcinoma.

| **ISUP grade group** | **N (TB + SB, reference)** | **TB alone, n (%)** | **TB alone, undiagnosed** | **TB + PB, n (%)** | **TB + PB, undiagnosed** |
| --- | --- | --- | --- | --- | --- |
| 1 (nsPCa) | 35 | 23 (65.7%) | 12 | 31 (88.6%) | 4 |
| 2 | 107 | 100 (93.5%) | 7 | 106 (99.1%) | 1 |
| 3 | 46 | 45 (97.8%) | 1 | 46 (100%) | 0 |
| 4 | 29 | 25 (86.2%) | 4 | 28 (96.6%) | 1 |
| 5 | 34 | 34 (100%) | 0 | 34 (100%) | 0 |
| csPCa total (grade group 2–5) | 216 | 204 (94.4%) | 12 | 214 (99.1%) | 2 |
